# Co-detection of measles virus vaccine strain and seasonal coronavirus in an immunocompromised patient with pneumonia

**DOI:** 10.1128/asmcr.00050-26

**Published:** 2026-05-21

**Authors:** C. Anderson, R. Odrobina, L. T. Murugan, B. T. Bradley

**Affiliations:** 1 Department of Pathology, University of Utah, Salt Lake City, Utah, USA; 2 ARUP Laboratories 14434, Salt Lake City, Utah, USA; 3 Division of Infectious Diseases, Department of Medicine, University of Utah 7060, Salt Lake City, Utah, USA; Rush University Medical Center, Chicago, Illinois, USA

**Keywords:** viral pneumonia, vaccine-preventable diseases, virology, measles

## Abstract

**Background:**

Measles vaccination is recommended for hematopoietic stem cell transplant (HSCT) recipients ≥24 months post-transplant in the absence of graft-versus-host disease and immunosuppressive therapy. Administration of live vaccines during immunosuppression may result in iatrogenic injury.

**Case Summary:**

We describe a 76-year-old patient with stage 3 multiple myeloma who had undergone autologous HSCT and received multiple therapies, including chimeric antigen receptor T-cell therapy and recent daratumumab treatment. Approximately 1 month after measles, mumps, and rubella (MMR) vaccination, she presented with progressive respiratory symptoms and hypoxia. Despite broad-spectrum antimicrobials and supportive care, her condition deteriorated, requiring ICU admission and mechanical ventilation. Molecular testing of her bronchoalveolar fluid detected the measles virus vaccine strain and seasonal coronavirus OC63. The patient succumbed to her illness on hospital day 16.

**Conclusion:**

This case highlights the rare but severe complication of measles vaccine-associated pneumonia in an immunocompromised host. While guidelines support MMR vaccination ≥24 months post-HSCT, ongoing immunosuppression may increase susceptibility to vaccine-related adverse events. Rising measles incidence in North America underscores the need for individualized vaccination strategies, balancing infection risk and vaccine safety. Healthcare providers should exercise caution when considering live-attenuated vaccines in HSCT recipients with recent immunosuppressive therapy. Further research is needed to refine vaccination guidelines for this vulnerable population.

## INTRODUCTION

Measles was eliminated from the U.S. nearly 30 years ago after sustained high two-dose coverage with the measles, mumps, and rubella (MMR) vaccine was achieved. Since then, vaccination rates have steadily decreased below the threshold for maintaining elimination. Disruptions caused by the COVID-19 pandemic and an upsurge in vaccine misinformation and hesitancy have been attributed to declining vaccination rates (1). As a result, the U.S. saw over 2,200 confirmed measles cases in 2025, the highest number since 1991. Many of these cases have been associated with large outbreaks in South Carolina, Texas, and Utah (2).

Hematopoietic stem cell transplant (HSCT) recipients are particularly vulnerable to measles due to the high transmissibility of the virus coupled with the patient’s reduced immunity. Current guidelines recommend MMR vaccination ≥24 months post-transplant, with the goal of protecting this high-risk population from severe community-acquired measles infection (3, 4). However, live-attenuated vaccines carry inherent risks for immunosuppressed individuals, and vaccine-associated disease—though rare—remains a documented complication in such settings (5).

## CASE PRESENTATION

**Fig 1:**
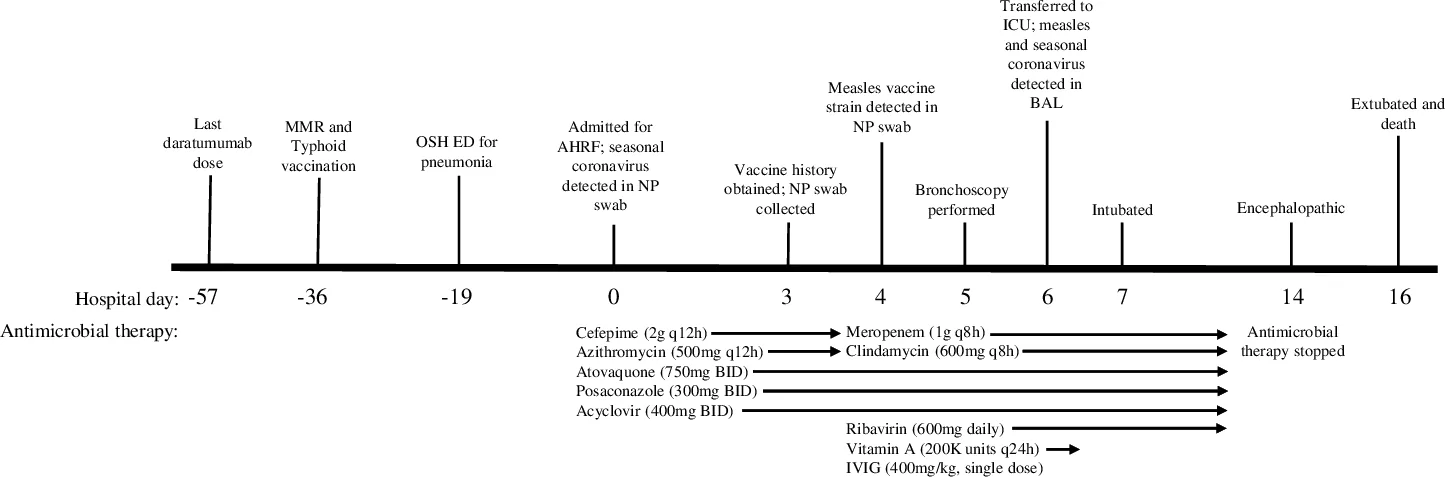
Case timeline and summary of antimicrobial therapy. AHRF, acute hypoxic respiratory failure; BAL, bronchoalveolar lavage; IVIG, intravenous immunoglobulin; MMR, measles, mumps, and rubella; NP, nasopharyngeal; OSH, outside hospital.

**Fig 2:**
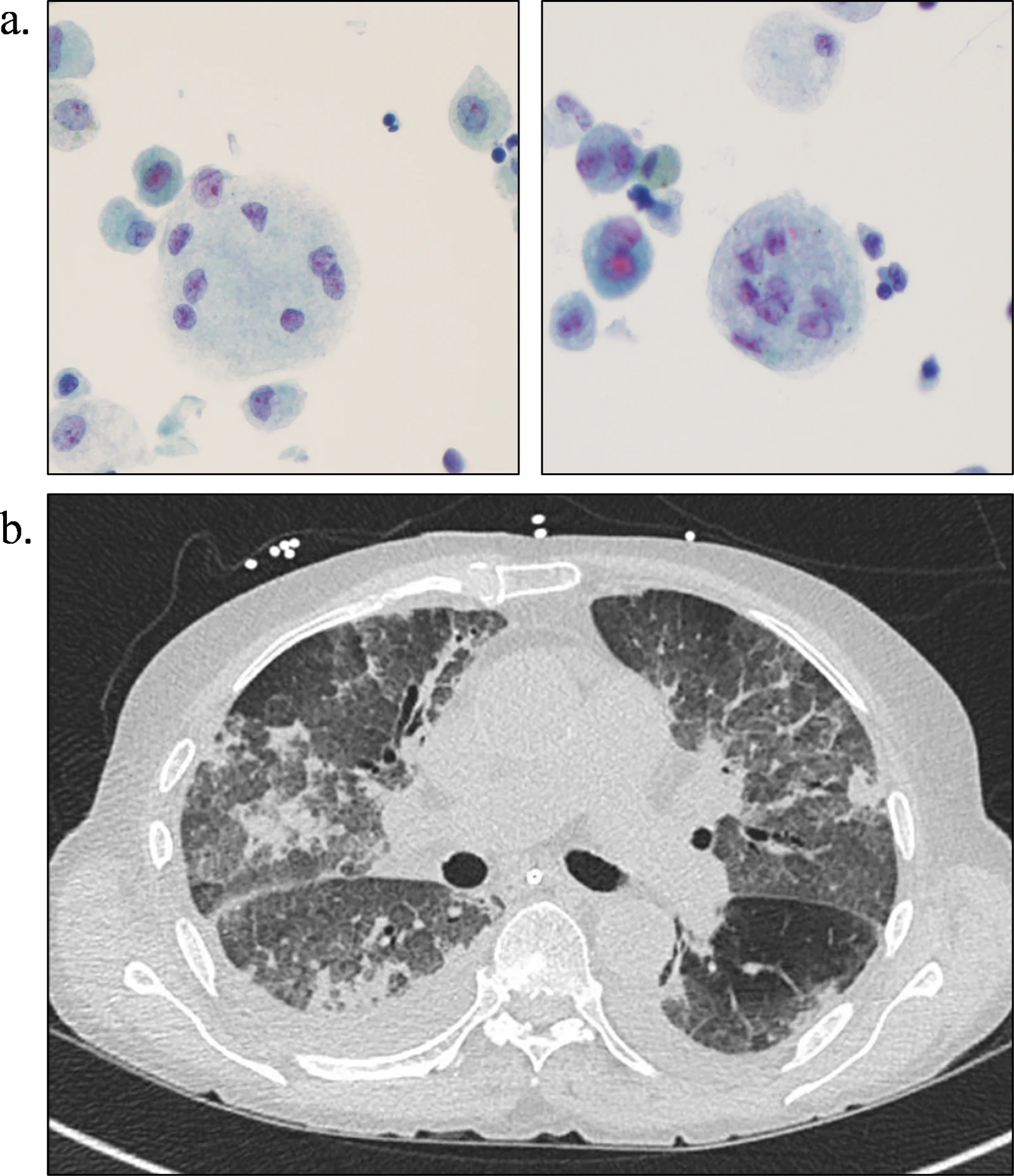
Cytology of bronchoalveolar lavage fluid and computed tomography of the chest. (**a**) Papanicolaou staining of bronchoalveolar lavage fluid demonstrating scattered multinucleated giant cells. Images taken at 400× magnification. (**b**) Computed tomography of the chest showing extensive bilateral bronchovascular ground-glass opacities and consolidations with diffuse bronchial wall thickening.

A 76-year-old female with a medical history significant for stage 3 multiple myeloma was transferred from the clinic to the inpatient service following concern for acute hypoxic respiratory failure and failure to thrive. The patient had received multiple lines of myeloma treatment, including autologous HSCT 10 years previously and chimeric antigen receptor T-cell therapy 2 years previously. Additionally, she had received daratumumab 2 months (day −57) before admission and was currently on intravenous immunoglobulin (IVIG) and prednisone therapy for Evans syndrome (Fig. 1). Three weeks (day −19) before admission to our institution, the patient presented to an outside emergency department and was treated for suspected community-acquired pneumonia after a computed tomography of the chest demonstrated left lower lobe consolidation. On admission to our institution (day 0), the patient endorsed extreme fatigue and shortness of breath requiring supplemental oxygen. She noted mild clinical improvement following the antibiotic course. At rest, her vital signs were within normal ranges, but upon exertion, she was tachypneic, hypoxemic, and required oxygen supplementation. Initial testing for infectious etiologies was significant for a nasopharyngeal swab positive for seasonal coronavirus (HCoV) OC43 via multiplex syndromic respiratory panel, indeterminate serum (1,3)-beta-D-glucan, negative nasal MRSA PCR, and negative urine pneumococcal antigen. Additional infectious workup was negative, including blood, respiratory, acid-fast bacillus, and fungal cultures; serum *Aspergillus* galactomannan; *Legionella pneumophila* urinary antigen; *Mucorales* PCR; *Coccidioides* IgG/IgM with immunodiffusion and complement fixation; cryptococcal antigen; and quantitative cytomegalovirus (CMV) and Epstein-Barr virus PCR. Chest CT demonstrated extensive bronchovascular ground-glass opacities and consolidation consistent with multifocal pneumonia. Other relevant findings included anemia (Hgb 9.8 g/dL, reference: 13.0–17.0 g/dL) with severe lymphopenia (30 cells/µL; 0.3%, reference: 1.30–3.60 k/µL and 17.6%–49.6%) and hypogammaglobulinemia (IgG 279 mg/dL; reference: 768–1,632 mg/dL). The patient received cefepime and azithromycin for empirical bacterial pneumonia coverage and acyclovir for CMV prophylaxis. Given a concern for atypical and opportunistic infections, the patient was also started on atovaquone for pneumocystis coverage due to a prior reaction to trimethoprim/sulfamethoxazole and fungal treatment with posaconazole. Upon further questioning, the patient reported receiving the MMR and typhoid polysaccharide vaccines in preparation for a trip to Africa approximately 1 month (day −36) before admission. Notably, her IgG was 123 mg/dL on the day of vaccination, and she had just decreased her prednisone taper from 20 to 10 mg/day. Due to the potential for measles vaccine-associated pneumonia, a nasopharyngeal swab was submitted for measles PCR testing at our institution using a laboratory-developed test that includes a vaccine-specific measles target (6). A positive measles virus vaccine strain detection (MeV-pan measles Ct: 30.4, MeVA-vaccine Ct: 30.6) was reported on hospital day 4. Subsequently, the patient was started on IVIG, vitamin A, and ribavirin. The patient was also switched to meropenem and clindamycin due to concern of superimposed bacterial pneumonia secondary to viral pneumonia. Two days later (day 6), the patient was transferred to the ICU due to increased oxygen requirements. A bronchoalveolar lavage performed on the previous day (day 5) detected both seasonal coronavirus via multiplex syndromic pneumonia panel and measles virus vaccine strain (MeV-pan measles Ct: 33.4, MeVA-vaccine Ct: 33.0). Cytology of the BAL was notable for scattered multinucleated giant cells compatible with viral pneumonia (Fig. 2a). Other BAL studies were negative, including bacterial, fungal, *Nocardia*, and AFB cultures; *Legionella* PCR; *Coccidioides* PCR; *Histoplasma*/*Blastomyces* PCR; *Aspergillus* PCR; *Aspergillus* galactomannan; and *Pneumocystis jirovecii* PCR. Additional negative infectious studies included *Coccidioides* serologies, cryptococcal antigen, and urine *Legionella* antigen. Despite broad-spectrum therapy, the patient continued to decline and was intubated on day 7. A repeat chest CT was notable for worsening multifocal infection (Fig. 2b). After progressive weakness and encephalopathy, the patient was transitioned to comfort care and succumbed to her illness on day 16.

## DISCUSSION

In this case report, we describe an immunocompromised individual who developed severe pneumonia with both HCoV and measles virus vaccine strain detected by PCR in a BAL specimen. Without histopathology and autopsy data, it is challenging to determine the relative contribution of each of these pathogens to the patient’s course as both have been associated with poor outcomes. Human CoV infections in immunocompromised individuals are associated with higher rates of invasive ventilation and mortality as compared to immunocompetent individuals (7–9). While there are fewer case reports of measles virus vaccine strain infections, severe outcomes have been documented in this population as well (5).

The prolonged PCR positivity of measles virus in this patient argues for a low degree of sustained viral replication. In healthy children who receive the MMR vaccine, PCR positivity was infrequently identified after 15 days (10). In our report, the patient tested positive at 39 and 41 days post-vaccination. In one extreme case, measles virus was detected 13 months after vaccination in an HIV patient who died of vaccine-associated measles pneumonitis (11).

The U.S. continues to struggle with a resurgence of measles with over 2,200 cases in 2025. The majority of cases have occurred in close-knit communities in Utah and South Carolina, with low vaccination coverage. Meanwhile, after 27 years, Canada has lost its elimination status as it continues to struggle with a protracted outbreak where cases have been reported in every province (12). As the U.S. approaches a similar milestone, immunocompromised individuals, especially HSCT recipients, are faced with an increasing risk of measles infection.

HSCT recipients are at a higher risk of progressive and severe measles infection due to a weakened immune system (13). After transplant, lymphocyte reconstitution may take up to 2 years, and most HSCT recipients will be seronegative for measles antibodies after transplantation (14, 15). Thus, MMR vaccination is the best strategy to protect HSCT recipients against measles infection. However, as with other live-attenuated vaccines, MMR vaccination is contraindicated in immunocompromised individuals, and guidelines generally consider live-attenuated vaccines to be safe 2 years after HSCT (16). Specifically, European and American guidelines advise administering the MMR vaccine to HSCT recipients beginning 24 months after transplantation, provided the patient has no graft-versus-host disease and is not on systemic immunosuppressive therapy (IST) or immunoglobulins (3, 4). One recommendation specifies ≥1 year off all systemic IST (17). These recommendations address the risk of developing vaccine-associated illness if HSCT recipients are vaccinated too soon after transplant although sooner vaccination may be considered in outbreak settings. A recent review of published cases found that vaccine-associated measles is a rare occurrence, and most cases were self-limiting and resolved without complications (5). Severe disease including measles inclusion body encephalitis, giant cell pneumonitis, and/or death was identified in three cases, all of which had an underlying immunocompromising condition. While immunocompromised individuals may develop severe symptoms, there has been no documented transmission of the measles virus vaccine strain between individuals. Similarly, current guidelines do not require these individuals to be placed on airborne precautions.

While the patient met society guidelines for MMR eligibility, it remains challenging to assess the relative contribution of her clinical findings (lymphopenia and hypogammaglobulinemia) and treatment (IVIG, daratumumab, and prednisone) to her overall immunosuppression and adverse outcome. IVIG prior to MMR vaccination may reduce vaccine efficacy but could potentially limit vaccine adverse events as well (18), whereas daratumumab, a CD38-targeting monoclonal antibody used to treat multiple myeloma, has been associated with an increased risk of viral infections (19, 20). While hypogammaglobulinemia and lymphopenia are recognized contributors to immunosuppression in multiple myeloma patients, the impact of daratumumab on adverse events related to live-attenuated vaccines remains unclear. A recent study evaluated the safety of MMR vaccination in patients with multiple myeloma receiving daratumumab after HSCT. Of the 41 patients included in the study, none developed an active measles infection after vaccination, and no hospitalizations or deaths were reported (21).

Given the complexity of immunosuppression and the relatively unknown impact of immunomodulatory therapies on vaccine responses, the decision to administer live-attenuated vaccines in HSCT recipients may require careful discussion between the patient and the healthcare provider. These complex decisions must account for the growing incidence of measles in the U.S. and balance the risks of vaccination versus the benefits of protection against community-acquired measles infection.
